# The synergistic effect of protein complex supplementation combined with 12 weeks of resistance training on isokinetic muscular function in untrained young males

**DOI:** 10.20463/jenb.2017.0036

**Published:** 2017-12-31

**Authors:** Jiwoong Jang, Hun-Young Park, Choongsung Yoo, Yeram Park, Jisu Kim, Kiwon Lim

**Affiliations:** 1. Physical Activity and Performance Institute (PAPI), Konkuk University, Seoul Republic of Korea; 2. Laboratory of Exercise Nutrition, Department of Physical Education, Konkuk University, Seoul Republic of Korea

**Keywords:** protein supplement, resistance training, body composition, inflammation, isokinetic

## Abstract

**[Purpose]:**

Resistance exercise training (RET) and an additional intake of dietary protein supplements may improve muscle mass and muscular function, and reduce inflammatory markers. The types, amount, and timing of dietary protein supplements are important for the synergistic effects of resistance training and dietary protein supplements. We hypothesized that a 25.1 g protein complex supplement taken for 12 weeks, immediately before and after resistance exercise, would enhance fat free mass and isokinetic muscular function in young untrained males.

**[Methods]:**

Eighteen participants were randomly assigned to a placebo (n=8) or protein complex supplement groups (n=10). The RET was a supervised progressive program, 3 times per week for 12-weeks, and was performed progressing 80% of their one repetition maximum (1-RM). Body composition, blood pressure, plasma inflammatory markers, lipid level and isokinetic muscular function were assessed before and after the study period.

**[Results]:**

There was a significant interaction effect in C-reactive protein (CRP) (*p* =0.044) among blood vessel inflammatory markers. The protein complex supplement group had shown more effective improvement at 12 weeks intervention compared to the placebo group in isokinetic muscular function. There was a significant interaction effect in peak torque at 60 degrees/sec leg extension (*p* =0.044), total work at 240 degrees/sec leg extension (*p* =0.025), and total work at 240 degrees/sec leg flexion (*p* =0.011).

**[Conclusion]:**

Protein complex supplementation during RET appears more effective than RET alone in improving isokinetic muscular function for 12 weeks in untrained young men.

## INTRODUCTION

Resistance exercise training (RET) has been generally considered a reliable strategy for improving muscle mass ^1^ and muscular function such as strength ^2^ and endurance ^3^. RET also is known to reduce inflammatory markers in blood vessels ^4^ and in muscle tissue ^5^. In addition, protein supplementation with resistance exercise has been reported to increase protein synthesis, resulting in improved muscular function ^6^^-^^7^. Therefore, RET combined with an additional intake of dietary protein supplements may be important to improve muscle mass and muscular function, and reduce inflammatory markers. 

Although there is a synergistic effect of additional protein supplementation on improving muscle size and strength through RET ^8^, this effect is not universal. In a recent study, resistance exercise (60 % to 80 % of a one-repetition maximum (1-RM), 3 times per week) and a protein blend (soy protein isolate, whey protein isolate and sodium caseinate; total 22 g), or a whey protein supplementation had no additional effect on 1-RM strength, isometric and isokinetic peak torque for flexion and extension, and whole-body lean body mass, over a 12 week period of RET in young men ^9^. Antonio et al. ^10^ reported that protein supplementation (average 18.3 g of essential amino acids [EAAs]) with a combined aerobic and resistance training program (20 min of aerobic exercise and 70% of 1-RM; 3 times per week) had no additional effects on measures of muscle mass and strength in untrained individuals. In contrast, Willoughby et al. ^11^ demonstrated that protein supplementation (14 g whey protein concentrate, 6 g whey protein isolate, 4 g milk protein isolate, 4 g calcium caseinate and 12 g of free amino acids; total 40 g) before and after resistance exercise (85 % to 90 % of 1-RM, 4 times per week) enhanced RET-induced fat free mass (FFM) and muscle strength in untrained individuals. As such, in untrained adults, it is difficult to conclude that protein supplementation during RET is more effective in increasing muscle mass and muscle strength. The differences in previous studies may be because of various factors, such as protein consumption timing, amount, and types, and resistance exercise intensity and duration. 

The types, amount, and timing of dietary protein supplements are important factors for synergistic effects in relation to resistance training and dietary protein supplements. Whey protein is divided into whey protein concentrates (WPCs), whey protein isolates (WPIs), and whey protein hydrolysates (WPHs). When a protein is hydrolyzed this means that it, through technological processes, it has been split into smaller chains of amino acids, called peptides. Therefore, WPHs are more rapidly absorbed and may be less allergenic than other forms of whey ^12^. Isotopic tracer studies have suggested that WPHs are more effective because of the higher digestion rate and the high branched chain amino acids (BCAAs) content than compared with other proteins such as micellar casein or soy protein isolate. A rapid digestion rate and a high BCAA content are two primary factors for increasing protein synthesis after resistance exercise ^13^. 

BCAAs are known to help increase muscle protein synthesis. Several studies have reported a minimal dose amount should comprise 5 g before and after resistance exercise to prevent resistance–related muscle fatigue, and to improve muscle function ^14^. The supplementation of pea protein with RET promoted gains in biceps brachii muscle thickness in humans ^15^, and led to reduced pro-inflammatory cytokines such as tumor necrosis factor-α (TNF-α) and interleukin-6 (IL-6) in mice ^16^. Therefore, blended type of whey protein, pea protein and, BCAA may be more effective in improving muscle strength and inflammation when combined with resistance exercise. 

In addition, the timing of protein supplementation is as important as the protein type. Candow et al. ^17^ suggested that taking the protein supplement immediately before or immediately after resistance exercise resulted in enhanced muscle hypertrophy, most like due to the increased blood flow and the rapid delivery of amino acids to the muscle tissue. It has also been reported that 20 g of protein consumption is effective for muscle protein synthesis after resistance exercise ^18^. 

Collectively, these studies demonstrate that the, type of protein supplement, the consumption timing immediately before and after RET, and the quantity of protein supplementation may all be important factors for improvement of muscle mass and muscular function. One meta-analysis has determined that protein supplementation enhance the gains in FFM and muscle strength after 3 months of prolonged RET ^8^. However, the effect of RET combined with a protein complex supplement consisting of whey protein, BCAA, and pea protein, (otherwise known to be effective in improving FFM, muscular function, and inflammation) has yet to be determined. Therefore, we hypothesized that a protein complex supplement comprising of WPCs, WPHs and pea protein (containing 4.9 g BCAAs) and an additional 1.1 g of BCAA, 1 g of creatine, 8 g of carbohydrate, and 1 g of fat before and after resistance exercise for 12 weeks, would enhance FFM and isokinetic muscular function in young untrained young males. 

## METHODS

### Participants and study design

Eighteen healthy young men (age, 24.0 ± 2.5 years; height, 175.1 ± 7.6 cm; weight, 73.4 ± 10.7 kg; mean ± S.D.) participated in the study. All participants did not participate in any other exercise except the exercise programs provided in this study and had no musculoskeletal disorders, and had no problems in participating in the various tests. Before the start of the study, we explained the study purpose and procedures, and obtained written consent from those who had understood and agreed to participate voluntarily. The protocol was approved by the Institution Review Board of the Konkuk University (Permit number: 7001355-2017-05-HR-176). Two participants in the placebo group voluntarily left the study because of muscular injury, and a total of eight participants completed 12 weeks of RET. The physical characteristics of the participants are shown in Table 1. 

**Table 1:** Participant characteristics before training.

	Placebo group (n=8)	Protein supplement group (n=10)
Age(y)	23.3 ± 3.1	24.6 ± 1.8
Weight(kg)	76.7 ± 10.3	70.8 ± 10.7
Height(cm)	174.6 ± 8.0	175.4 ± 7.8
BMI(kg/m^2^)	25.1 ± 2.6	22.6 ± 2.9
Fat free mass(kg)	58.9 ± 6.7	58.1 ± 6.3
Fat mass(kg)	17.8 ± 4.8	12.7 ± 6.5

Note. Data are means ± S.D. BMI = body mass index

### RET protocol

The RET was a supervised progressive program that, occurred 3 times a week for 12 weeks. Three sets 10-12 repetition exercise were performed, with a 60 sec rest period between sets to prevent muscle fatigue. The program consisted of lower body exercises (barbell squat, dead lift, seated leg extension, and lying leg curl), and upper body exercises (bench press, barbell rowing, preacher bench biceps curl, and dumbbell shoulder press). Resistance exercise sessions lasted approximately 45 min, with a 10 min warm-up and cool-down session performed before and after resistance exercise. All sessions were supervised by a physical trainer. The participants performed the resistance training progressing 80 % of their 1-RM. The 1-RM was assessed at baseline and was re-measured every 4 weeks. The 1-RM measurement was indirectly estimated through the repetition of the submaximal load. After a warm-up period, all the participants lifted a submaximal load until exhaustion (10 to 12 RM). The 1-RM was predicted using the Brzycki equation: predicted 1-RM = weight lifted (kg) / [1.0278 – (number of repetitions × 0.0278)] ^19^. On the days when no training, the participants did not perform any additional exercise. The resistance exercise program is shown in Table 2. 

**Table 2:** The 12-weeks resistance exercise program

Type	Content		Intensity	Time
Warm- up	Stretching		RPE 8 to10	10 min
Main Exercise	Upper	Bench press	3sets 10 to 12 rep/set Rest : 60sec	45 min
		Barbell rowing		
		Preacher bench biceps curl		
		Dumbbell shoulder press		
	Lower	Barbell squat		
		Dead lift		
		Seated leg extension		
		Lying leg curl		
Cool- down	Stretching		RPE 8 to10	10 min

### Supplementation

The participants were equally assigned to a placebo or protein supplement groups. The protein supplement group consumed 40 g of a blended protein powder (24 g of whey protein concentrates, hydrolyzed whey protein and pea protein; containing 4.9 g BCAA, 1.1 of BCAAs, 1g of creatine, 8 g of carbohydrate, 1g of fat and flavoring and sweeteners). The placebo group consumed the supplement minus the protein, BCAA, and the creatine components. On training days, the participants each received one supplement sachet dissolved in 250 ml, to drink immediately before training, and another equivalent sachet immediately after the training session. On the days when no training occured, the participants consumed their regular food without additional supplementation. 

### Body composition

Body composition was measured every 4 weeks using an X-Scan plus II (Jawon Medical, Siheung, Korea) body composition analyzer to determine the body composition changes over the 12 weeks of RET. Prior the body composition measurement, all participants were required to fast for a minimum of 8 hours. 

### Blood pressure

Blood pressure was measured every 4 weeks using an automated blood pressure monitor (JPN 1, Omron Healthcare, Kyoto, Japan) to determine the change in systolic blood pressure (SBP), diastolic blood pressure (DBP), and mean arterial blood pressures (MAP) through the 12 weeks of RET. Blood pressure was measured following a 10-minute rest period. The SBP, DBP and MAP were recorded as an average of two readings. 

### Isokinetic muscular function test

Unilateral isokinetic muscle function of the knee and the elbow were measured using a Biodex system 4 Pro isokinetic dynamometer (Biodex Medical Systems, Shirley, NY, USA). The participants were fully informed regarding the test method and procedures prior to the measurement. During the knee test, the participants were seated and the lateral epicondyle of the knee joint was aligned with the dynamometer lever arm. The contact pad was placed approximately 3 cm superior to the medial malleolus, with the foot placed in a plantigrade position. The range of motion (ROM) was set from 90^o^ knee flexion to 0^o^ extension. The gravity effect torque was measured to exclude the effect of the weight of the lower limb on muscle strength. During the elbow test, the chair was rotated to 90^o^ and the dynamometer was rotated 30^o^ outward from the chair. The elbow axis (lateral epicondyle of humerus) was aligned to that of the dynamometer. The ROM for the elbow test was set 130^o^ elbow flexion to 0^o^ extension. To stabilize the participants on the chair, a stabilization belt was fastened across the chest, and a Velcro arm strap was attached around the upper arm to keep the arm against the elbow axis of rotation in the correct position. The knee and elbow tests were both conducted at 60 degrees/sec and at 240 degrees/sec to assess maximum muscle strength and muscle endurance, respectively. 

### Blood sampling and biochemical analysis

Blood samples were obtained from the antecubital vein 48h after a training session throughout the study, using appropriate collection tubes (Vacutainer, Beckton Dickinson, Rutherford, NJ, USA). Blood samples were centrifuged at 3,000 rpm for 15min at 4℃ for serum and plasma isolation in order to perform biochemical analysis. A Cobas 8000 Modular Analyzer (Roche Diagnostics, Manheim, Germany) was used to obtained serial C-reactive protein (CRP), lactate dehydrogenase (LDH), creatine kinase (CK), total cholesterol (TC), low-density lipoprotein (LDL), and high-density lipoprotein (HDL) levels. The analysis was conducted by Green Cross Corporation, in Korea. 

### Data analysis

All analyses were performed using the Statistical Package for Social Science (SPSS) version 23.0 (IBM Corp., Armonk, NY, USA). Data are presented as means ± standard deviations (S.D.). Kolmogorov-Smirnov tests were used to ensure that all data had a normal distribution. Twoway ANOVA with repeated measures (Time [0, 4, 8, and 12 weeks] × Supplement [placebo and protein complex supplement]), followed by Bonferroni post hoc comparisons, were performed. Partial eta-squared (η^2^) was calculated as a measures of effect size. The level of significance was set at 0.05. 

## RESULTS

### Body composition

The pre and post-intervention data for all body composition parameters, showed no significant interaction between the placebo and the protein complex supplement groups. However, there was a significant effect within the timeframe in respect of FFM (*p* =0.003, η^2^=0.275), therefore, resistance training appears to be effective in increasing FFM (Table 3). 

**Table 3:** Change in body composition for 12 weeks training in each supplement group

Variable	Group	Training period (week)					*p*	η^2^
		0	4	8	12			
Weight (kg)	Pla	76.7 ± 0.3	76.1 ± 9.6	75.9 ± 8.8	77.4 ± 8.9	Ex Suppl Ex x Suppl	0.209 0.258 0.830	0.089 0.079 0.018
	Pro	70.8 ± 10.7	71.2 ± 9.6	71.2 ± 9.1	72.3 ±8.6			
FFM (kg)	Pla	58.9 ± 6.7	59.3 ± 6.0	58.5 ± 5.7	60.3 ± 6.2	Ex Suppl Ex x Suppl	0.003^††^ 0.996 0.453	0.275 0.000 0.050
	Pro	58.1 ± 6.3	59.8 ± 6.7	58.9 ± 6.1	60.3 ± 6.7			
FFM (kg)	Pla	17.8 ± 4.8	16.8 ± 5.4	17.5 ± 4.9	17.1 ± 4.8	Ex Suppl Ex x Suppl	0.132 0.057 0.824	0.116 0.208 0.003
	Pro	12.7 ± 6.5	11.4 ± 6.2	12.3 ± 5.0	12.1 ± 5.4			

Note. Data are means ± S.D. Pla: placebo group, Pro: protein supplement group, Ex: exercise, Suppl: supplement, FFM: fat free mass, FM: fat mass.

^††^Significant main effect, *p* <0.01.

### Blood pressure

No significant interaction was observed in all blood pressure variables. However, there was a significant effect within the time frame in respect of SBP (*p* =0.003, η^2^=0.284), DBP (*p* <0.001, η^2^=0.598), and MAP (*p* <0.001, η^2^=0.559). Therefore, resistance training appears to be effective in improving SBP, DBP, and MAP (Table 4). 

**Table 4:** Change in blood pressure for 12 weeks training in each supplement group

Variable	Group	Training period (week)					*p*	η^2^
		0	4	8	12			
SBP (mmHg)	Pla	127.0 ± 13.6	125.6 ± 14.7	126.2 ± 10.4	120.3 ± 10.8	Ex Suppl Ex x Suppl	0.003^††^ 0.169 0.405	0.284 0.15 0.056
	Pro	122.7 ± 7.2	115.8 ± 7.3	122.2 ± 7.7	114.5 ± 8.4			
DBP (mmHg)	Pla	75.5 ± 8.7	67.9 ± 9.7	68.1 ± 8.8	65.5 ± 6.3	Ex Suppl Ex x Suppl	0.000^††^ 0.096 0.604	0.598 0.163 0.036
	Pro	70.8 ± 8.4	62.2 ± 7.4	63.1 ± 7.2	57.4 ± 6.7			
MAP (mmHg)	Pla	92.7 ± 9.9	87.1 ± 11.0	87.4 ± 9.0	83.7 ± 7.2	Ex Suppl Ex x Suppl	0.000^††^ 0.099 0.577	0.559 0.160 0.037
	Pro	88.1 ± 7.6	80.1 ± 6.5	82.8 ± 6.3	76.4 ± 5.8			

Note. Data are means ± S.D. Pla: placebo group, Pro: protein supplement group, Ex: exercise, Suppl: supplement, SBP: systolic blood pressure, DBP: diastolic blood pressure, MAP: mean arterial pressure.

^††^Significant main effect, *p*<0.01.

^†††^Significant main effect, *p*<0.001.

### Plasma inflammation markers

As shown in Table 5, there was a significant interaction in CRP (*p* =0.044, η^2^=0.170), however, no significant interaction was observed in CK and LDH. 

**Table 5:** Change in blood vessel and muscle inflammation factors for 12 weeks training in each supplement group

Variable	Group	Training period (week)					*p*	η^2^
		0	4	8	12			
CRP (mg/dl)	Pla	0.03 ± 0.03	0.05 ± 0.06	0.11 ± 0.11	0.05 ± 0.02	Ex Suppl Ex x Suppl	0.066 0.924 0.044^†^	0.150 0.001 0.170
	Pro	0.07 ± 0.10	0.06 ± 0.08	0.06 ± 0.06	0.05 ± 0.03			
CK (U/l)	Pla	176.6 ± 124.8	127.3 ± 50.4	166.4 ± 33.9	223.1 ± 58.4	Ex Suppl Ex x Suppl	0.024^†^ 0.485 0.534	0.208 0.031 0.038
	Pro	165.1 ± 65.4	172.4 ± 60.4	194.8 ± 60.4	228.7 ± 108.2			
LDH (U/l)	Pla	318.0 ± 30.9	307.5 ± 34.6	354.9 ± 37.3	346.6 ± 41.3	Ex Suppl Ex x Suppl	0.000^†††^ 0.370 0.634	0.467 0.051 0.029
	Pro	296.7 ± 32.6	297.4 ± 40.2	333.2 ± 37.0	341.7 ± 56.2			

Note. Data are means ± S.D. Pla: placebo group, Pro: protein supplement group, Ex: exercise, Suppl: supplement, CRP: C-reactive protein, CK: creatine kinase, LDH: lactate dehydrogenase

^†^Significant interaction or main effect, *p* <0.05.

^†††^Significant interaction or main effect, *p* <0.001.

### Blood lipid level

No significant interaction was observed in TC, LDL-C, and HDL-C. However, there was a significant effect within the time frame in respect of TC (*p* =0.036, η^2^=0.178) and HDL-C (*p* <0.001, η^2^=0.540). Therefore, resistance training appears to be effective in improving TC and HDL-C (Table 6). 

**Table 6:** Change in blood lipid level for 12 weeks training in each supplement group

Variable	Group	Training period (week)					p	η^2^
		0	4	8	12			
TC (mg/dl)	Pla	180.5 ± 38.5	165.5 ± 28.1	183.8 ± 33.3	179.0 ± 26.9	Ex Suppl Ex x Suppl	0.036^†^ 0.784 0.366	0.178 0.005 0.062
	Pro	176.4 ± 42.0	177.7 ± 40.9	188.2 ± 35.6	184.2 ± 33.6			
LDL (mg/dl)	Pla	108.9 ± 36.2	91.4 ± 24.9	104.6 ± 24.1	100.4 ± 25.4	Ex Suppl Ex x Suppl	0.135 0.503 0.326	0.113 0.029 .068
	Pro	110.2 ± 36.1	108.6 ± 31.2	112.4 ± 29.0	109.6 ± 24.5			
HDL (mg/dl)	Pla	56.9 ± 10.1	61.4 ± 12.0	68.9 ± 14.8	66.3 ± 13.1	Ex Suppl Ex x Suppl	0.000^†††^ 0.316 0.268	0.540 0.063 0.079
	Pro	52.2 ± 12.8	52.1 ± 10.0	62.1 ± 13.6	64.2 ± 13.1			

Note. Data are means ± S.D. Pla: placebo group, Pro: protein supplement group, Ex: exercise, Suppl: supplement, TC: total cholesterol, LDL: low density lipoprotein, HDL: high density lipoprotein.

^†^Significant main effect, *p* <0.05.

^†††^Significant main effect, *p* <0.001.

### Isokinetic muscular function

Tables 7-10 depicts pre- and post-intervention data for isokinetic muscular function parameters in both groups. There was a significant interaction in peak torque at 60 degrees/sec leg extension (*p* =0.044, η^2^=0.154), total work at 240 degrees/sec leg extension (*p* =0.025, η^2^=0.176), and total work at 240 degrees/sec leg flexion (*p* =0.011, η^2^=0.249) among the isokinetic muscular function parameters. 

**Table 7:** Change in upper extremity isokinetic muscular function parameters at 60 degrees/sec for 12 weeks training in each supplement group

Variable		Group	Training period (week)					*p*	η^2^
			0	4	8	12			
Ext	Peak torque (Nm)	Pla	53.8 ± 7.7	54.8 ± 5.3	57.8 ± 5.3	58.8 ± 3.9	Ex Suppl Ex x Suppl	0.000^†††^ 0.627 0.111	0.585 0.015 0.134
		Pro	53.3 ± 10.8	57.5 ± 9.9	58.9 ± 10.6	63.3 ± 10.7			
	Peak torque / BW (%)	Pla	71.6 ± 8.3	75.0 ± 6.9	77.3 ± 6.1	91.2 ± 18.8	Ex Suppl Ex x Suppl	0.000^†††^ 0.207 0.081	0.649 0.098 0.152
		Pro	75.8 ± 16.8	83.6 ± 16.9	86.1 ± 16.7	40.1 ± 5.3			
Flex	Peak torque (Nm)	Pla	34.6 ± 4.4	39.4 ± 5.6	38.4 ± 4.7	40.1 ± 5.3	Ex Suppl Ex x Suppl	0.000^†††^ 0.408 0.453	0.661 0.043 0.048
		Pro	32.0 ± 4.7	38.0 ± 5.3	35.9 ± 4.9	39.3 ± 4.3			
	Peak torque / BW (%)	Pla	45.8 ± 7.6	52.0 ± 7.0	50.9 ± 6.3	52.0 ± 6.5	Ex Suppl Ex x Suppl	0.000^†††^ 0.711 0.361	0.634 0.009 0.063
		Pro	46.1 ± 9.4	54.3 ± 11.2	51.3 ± 10.4	55.1 ± 9.3			

Note. Data are means ± S.D. Pla: placebo group, Pro: protein supplement group, Ex: exercise, Suppl: supplement, Ext = extension, Flex = flexion.

^†††^Significant main effect, *p* <0.01.

As a results, Bonferroni post hoc comparisons were performed, and peak torque at 60 degrees/sec leg extension, in the placebo group showed no significant difference between the time points. In the protein complex supplement group presented a significantly higher value at 12 weeks compared with week 0 (*p* <0.05), 4 weeks (*p* <0.05), and 8 weeks (*p* <0.05) (Table 8). 

**Table 8:** Change in lower extremity isokinetic muscular function parameters at 60 degrees/sec for 12 weeks training in each supplement group

Variable		Group	Training period (week)					*p*	η^2^
			0	4	8	12			
Ext	Peak torque (Nm)	Pla	200.8 ± 21.2	189.8 ± 9.2	201.4 ± 16.4	201.6 ± 22.7	Ex Suppl Ex x Suppl	0.000^†††^ 0.681 0.044^†^	0.320 0.011 0.154
		Pro	201.9 ± 45.1	200.8 ± 43.0	202.0 ± 38.4	214.7 ± 39.1^a^^,^^b^^,^^c^			
	Peak torque / BW (%)	Pla	264.6 ± 26.3	253.4 ± 29.4	267.1 ± 181	265.5 ± 32.3	Ex Suppl Ex x Suppl	0.031† 0.186 0.110	0.178 0.107 0.122
		Pro	287.3 ± 56.0	285.9 ± 53.9	283.2 ± 45.5	299.8 ± 44.2			
Flex	Peak torque (Nm)	Pla	93.0 ± 11.7	93.7 ± 9.0	102.2 ± 20.1	103.0 ± 18.0	Ex Suppl Ex x Suppl	0.000^†††^ 0.949 0.653	0.389 0.000 0.028
		Pro	91.9 ± 24.2	94.7 ± 17.6	100.5 ± 25.0	106.9 ± 20.0			
	Peak torque / BW (%)	Pla	122.6 ± 14.2	124.8 ± 15.6	134.7 ± 18.0	139.0 ± 16.4	Ex Suppl Ex x Suppl	0.000^†††^ 0.398 0.894	0.399 0.045 0.011
		Pro	131.3 ± 32.8	135.7 ± 27.3	142.7 ± 36.8	151.6 ± 29.9			

Note. Data are means ± S.D. Pla: placebo group, Pro: protein supplement group, Ex: exercise, Suppl: supplement, Ext: extension, Flex: flexion.

^†^Significant interaction or main effect, *p* <0.05.

^††^Significant main effect, *p* <0.01.

^†††^Significant main effect, *p* <0.01.

^a^* p* <0.05 versus. Week0

^b^* p* <0.05 versus. Week4

^c^* p* <0.05 versus Week8

**Table 9:** Change in upper extremity isokinetic muscular function parameters at 240 degrees/sec for 12 weeks training in each supplement group

Variable		Group	Training period (week)					*p*	η^2^
			0	4	8	12			
Ext	Average power (watts)	Pla	71.6 ± 6.4	76.2 ± 9.3	82.1 ± 11.8	79.9 ± 10.4	Ex Suppl Ex x Suppl	0.002^†††^ 0.578 0.129	0.322 0.020 0.120
		Pro	74.2 ± 18.9	78.2 ± 19.3	83.3 ± 19.1	91.2 ± 25.8			
	Total work (J)	Pla	1050.5 ± 51.9	1128.2 ± 77.9	1176.4 ± 56.0	1141.2 ± 103.4	Ex Suppl Ex x Suppl	0.000^†††^ 0.773 0.111	0.383 0.005 0.130
		Pro	1040.4 ± 237.7	1136.7 ± 225.1	1170.5 ± 231.7	1253.0 ± 322.1			
Flex	Average power (watts)	Pla	22.2 ± 4.5	28.1 ± 4.7	28.4 ± 6.5	28.9 ± 5.4	Ex Suppl Ex x Suppl	0.000^†††^ 0.833 0.446	0.550 0.003 0.045
		Pro	21.9 ± 4.8	27.5 ± 5.3	28.9 ± 4.2	30.9 ± 4.6			
	Total work (J)	Pla	360.3 ± 56.9	419.6 ± 97.7	422.7 ± 94.5	424.6 ± 109.4	Ex Suppl Ex x Suppl	0.000^†††^ 0.935 0.314	0.592 0.000 0.067
		Pro	339.9 ± 58.6	414.7 ± 48.1	433.7 ± 58.8	449.2 ± 58.4			

Note. Data are means ± S.D. Pla: placebo group, Pro: protein supplement group, Ex: exercise, Suppl: supplement, Ext: extension, Flex: flexion.

^††^Significant main effect, *p* <0.01.

^†††^Significant main effect, *p* <0.01.

For the total work at 240 degrees/sec leg extension, the placebo group showed a significantly higher value at 12 weeks than at week 0 (*p* <0.05) and at 4 weeks (*p* <0.05), and the protein complex supplement group presented a significantly higher value at 4 weeks than at week 0 (*p* <0.05). There was a significant increase at 12 weeks compared with week 0 (*p* <0.05), 4 weeks (*p* <0.05), and 8 weeks (*p* <0.05) in the protein supplement group. The increased rate, via the intervention, showed a higher value in the protein complex supplement group than in the placebo group (Table 10). 

**Table 10:** Change in lower extremity isokinetic muscular function parameters at 240 degrees/sec for 12 weeks training in each supplement group

Variable		Group	Training period (week)					*p*	η^2^
			0	4	8	12			
Ext	Average power (watts)	Pla	185.8 ± 32.8	182.3 ± 25.7	193.0 ± 26.5	195.5 ± 24.6	Ex Suppl Ex x Suppl	0.002^†††^ 0.966 0.080	0.322 0.000 0.147
		Pro	176.4 ± 29.7	190.3 ± 30.3	186.6 ± 26.5	205.6 ± 31.1			
	Total work (J)	Pla	1764.9 ±287.8	1770.2 ± 232.6	1871.2 ± 236.7	1892.6 ± 231.1 ^a^^,b^	Ex Suppl Ex x Suppl	0.000^†††^ 0.818 0.025†	0.383 0.003 0.176
		Pro	1694.2 ± 266.7	1891.4 ± 309.1^a^	1814.5 ± 295.3	2015.8 ± 354.7 ^a^^,b,c^			
Flex	Average power (watts)	Pla	83.0 ± 27.0	79.2 ± 28.0	88.9 ± 31.0	95.7 ± 35.0	Ex Suppl Ex x Suppl	0.000^†††^ 0.645 0.060	0.550 0.014 0.164
		Pro	79.1 ± 21.0	90.1 ± 24.8	93.7 ± 26.1	107.4 ± 27.9			
	Total work (J)	Pla	860.7 ± 270.9	876.6 ± 259.6	934.8 ± 293.5 ^a^^,b^	962.0 ± 311.9 ^a^^,b^	Ex Suppl Ex x Suppl	0.000^†††^ 0.237 0.011^†^	0.592 0.086 0.249
		Pro	893.9 ± 202.8	1030.9 ± 235.0 ^a^	1084.9 ± 216.0 ^a^	1190.0 ± 228.5 ^a^^,b,c^			

Note. Data are means ± S.D. Pla: placebo group, Pro: protein supplement group, Ex: exercise, Suppl: supplement, Ext: extension, Flex: flexion.

^†^Significant interaction effect, *p*<0.05.

^††^Significant main effect, *p*<0.01.

^†††^Significant main effect. *p*<0.01.

^a^* p* <0.05 versus. Week0, η^2^*p* <0.05 versus. Week4, η^2^*p* <0.05 versus Week8.

For the total work at 240 degrees/sec leg flexion, the placebo group showed a significantly higher value at 8 weeks (*p* <0.05) and at 12 weeks (*p* <0.05) than at week 0 and 4weeks, respectively. The protein complex supplement group presented a significantly higher value at 12 weeks than at week 0 (*p* <0.05), 4 weeks (*p* <0.05), and 8 weeks (*p* <0.05). There was a significant increase at 4 weeks (*p* <0.05) and at 8 weeks (*p* <0.05) compared with 0 week (Table 10). The increase rate for 12 weeks involving all isokinetic muscular function parameters with significant interaction (exercise × supplement) showed a higher value in the protein complex supplement group than in the placebo group. 

## DISCUSSION

In this study, we confirmed the synergistic effect of pre- and post-RET consumption of a 25.1 g protein complex supplement compared to a placebo group on isokinetic muscular function and CRP during 12 weeks of RET in untrained young men. However, the protein complex supplement combined with RET group demonstrated a tendency increase in FFM, but there was no interaction effect. These results showed that RET combined with a supplement protein complex improved in CRP and isokinetic muscular function more than RET alone. 

The protein supplement group showed a tendency to increase FFM more than the placebo group, but there was no statistically significant difference. However, unlike our results, Andersen et al. ^20^ reported that consumption of protein supplements immediately before and after resistance exercise significantly increased FFM after 10 weeks of training, compared to a placebo group. The reason for the difference in the change of FFM between the present study and Andersen et al.’s study ^20^ may possibly be due to the difference in intensity (85 % to 90 % versus 80 % of 1-RM), the duration of the resistance exercise (14 weeks versus 12 weeks) and the frequency of the protein supplement intake (daily intake versus intake only on exercise days). As a result, the differences in exercise intensity and frequency, and the differences in the amount of protein supplements consumed during the training period may explain the difference in FFM changes. 

Dual-energy X-ray absorptiometry (DEXA), computed tomography (CT), magnetic resonance imaging (MRI), and bioelectrical impedance analysis (BIA) are used to measure body composition or muscle mass. While the accuracy of DEXA, MRI and CT is well recognized, the accuracy of BIA is controversial. BIA depends largely on precise application of the instrumentation involving measurement factors such as temperature, humidity, and condition of the skin ^21^. Therefore, to measure muscle mass more accurately, it is recommended that CT, DEXA, or MRI be used to accurately visualize the cross-sectional area of muscle. However, due to research costs and availability, our study used the BIA method to measure FFM, and this is a limitation of the study. 

Some studies have suggested that protein supplement ^22^^-^^23^ or resistance exercise ^24^ interventions can be positive factors in blood pressure control. Therefore, we considered that RET combined with protein supplement may have a synergistic effect in improving blood pressure. In our results, SBP decreased 6.7 mmHg and 8.2 mmHg in the placebo and protein supplementation group, respectively, and showed a 10 mmHg and 13.4 mmHg decrease in DBP, respectively. The protein supplement groups tended to show a larger decrease in SBP and DBP than the placebo group, but there was no synergistic effect between RET and the protein supplement. These results suggest that RET combined with a protein complex did not demonstrate a synergistic effect in positively regulating blood pressure. While the RET combined with the protein complex supplement did not show a synergistic effect in improving blood pressure, this is probably because the participants blood pressure was within a normal range in both the protein supplement and the placebo group. 

Whey protein supplementation has beneficial effect on reducing the circulating CRP level. This CRP level is a haematological marker for inflammation that is synthesized in the liver in response to cytokines release by damaged tissue ^25^. In this study, there was a significant interaction in CRP (*p* =0.044). CRP increased 0.02 mg/dl in the placebo group but decreased 0.02 mg/dl in the protein supplement group. Although the type of protein consumed was different, Pins et al. ^26^ reported a significant decrease in CRP level after six consecutive weeks of hydrolyzed whey protein consumption, which was consistent with our result. Ndiate et al. ^16^ reported that pea protein reduced pro-inflammatory cytokines, and TNF-α and IL-6, between 35 % and 80 %, respectively. However, IL-6, a pro-inflammatory marker known to induce CRP expression was not measured in this study, and IL-6 inhibition by the pea protein might have affected the CRP level. Previous studies have reported that RET reduced blood CRP levels, and most of these reports involved older or over-weight participants ^27^^-^^28^. In previous studies, the reason why resistance exercise reduced blood CRP levels was considered to be because of decreased body fat or increased muscle mass induced through RET. However, it is unclear how whey protein and RET affects the CRP level, and further study is required to determine this. 

Resistance exercise can result in localized damage to muscle tissue. Plasma CK and LDH levels are indicators of muscle damage after resistance exercise. In this study, plasma CK and LDH levels showed a significant increase at 12 weeks compared to week 0, which is considered a natural phenomenon due to RET, and these results are consistent with previous studies. Rodrigues et al. ^29^ reported that CK and LDH increased after 3 days of resistance exercise. 

DeNysschen et al. ^23^ reported that RET combined with a soy or whey protein supplement taken over a 12 week period (3 times per week) did not affect the blood lipid levels compared to the placebo group, in overweight and obese individuals. Similar to the previous study, there was no significant change in TC, LDL, and HDL cholesterol in both groups in this study. These results suggest that RET combined with a protein complex supplement did not appear to show a synergistic effect in regulating blood pressure positively, nor did it show a synergistic effect in improving blood lipid levels. This is most likely because the participants blood lipid levels were within the normal range in both the protein supplement group and in the placebo group. 

RET is generally considered a reliable strategy for improving muscular function such as strength ^2^ and endurance ^3^. In this study, RET combined with protein complex supplement had a synergistic effect on muscle strength and endurance on the lower extremity. An isokinetic muscular function, angular velocity 60 degrees/ sec test was used as an index of muscle strength, and 240 degrees/sec test was used as an index of muscle endurance ^30^. There was a significant interaction in peak torque at 60 degrees/sec leg extension, total work at 240 degrees/second leg extension, and total work at 240 degrees/sec leg flexion among isokinetic muscular function parameters in this study. These results are consistent with previous RET study results concerning an increase in muscle strength and endurance. 

Previous studies have shown that protein supplementation significantly increased muscle strength compared to placebo groups. According to Candow et al.’s ^31^ results, muscular strength was significantly increased in both upper and lower extremities after 6 weeks of RET in a group that consumed whey (1.2 g/kg body mass whey protein + 0.3 g/kg body mass sucrose power) compared with a placebo group with no protein intake. Burke et al. ^32^ reported that peak torque on knee extension after a 12 week RET in a group consuming mixed whey protein and creatine (1.2 g/kg/day whey protein + 0.1 g/kg/day creatine) was significantly increased compared to the placebo group. In our study, as in Burke et al. ^32^, the consumption of protein and creatine in a blended form (25.1 g protein + 1 g creatine), and similar to a previous study, increased knee extension peak torque. However, in our study, the increase in knee extension peak torque is not considered to be an effect of creatine consumption because of the difference in the amount of creatine intake. In our study, participants consumed a total of 2 g creatine before and after RET, and in the Burke et al. study the participants consumed approximately 8 g of creatine were consumed. Other studies have reported that a sufficient amount (5 g to 20 g) of creatine intake is effective in improving muscular function ^33^^-^^35^. Therefore, it is unlikely that a total of 2 g creatine intake affected the increase of knee extension peak torque. Although direct comparisons are difficult, the results of previous studies and our study suggest that protein complex supplementation or a single type of protein supplement may have similar effects in improving muscle strength. 

In this study, RET combined with protein complex supplement increased the knee extension and flexion isokinetic total work. Consistent with our study, Selig et al. ^3^ also reported that resistance exercise increased the knee isokinetic muscle endurance of the knee. The knee total work significantly increased in both the protein supplement and placebo groups after 12 weeks compared with pre RET interventions. In particular the protein supplement group tended to increase in both extension (protein complex supplement group: 19 %, placebo group: 7.2 %) and flexion (protein complex supplement group: 33.1 %, placebo group: 11.8 %). Therefore, RET combined with protein complex supplement was more effective than RET alone in increasing muscle endurance. 

The protein complex supplement group tended to increase upper extremity strength and endurance more than in the placebo group, but there was no synergistic effect. Although the reason is unclear, the difference in muscle mass change between the upper and lower extremities seems to have induced these results. Therefore, it is necessary to compare the results with accurate measurement of muscle mass of both the upper and lower extremities using CT or MRI. 

In the present study, there was a limitation in that the participant’s daily food intake could not be controlled, and the number of participants was small. Also, CT and MRI would provide more accurate measurement of changes to muscle mass than the BIA method. Further research that increases the frequency of protein supplement intake and convers a longer training period is required. 

In conclusion, while the results of this study did not fully support RET combined with protein complex supplement in providing a significant synergistic effect on lean body mass and on inflammatory markers, there was a synergistic effect in improving isokinetic muscle strength and endurance in untrained young men. 
